# Current Status of Genetically Modified Pigs That Are Resistant to Virus Infection

**DOI:** 10.3390/v14020417

**Published:** 2022-02-17

**Authors:** Hongming Yuan, Lin Yang, Yuanzhu Zhang, Wenyu Xiao, Ziru Wang, Xiaochun Tang, Hongsheng Ouyang, Daxin Pang

**Affiliations:** Key Laboratory of Zoonosis Research, Ministry of Education, Jilin Provincial Key Laboratory of Animal Embryo Engineering, Department of Biotechnology, College of Animal Sciences, Jilin University, Changchun 130062, China; yuanhongming@jlu.edu.cn (H.Y.); yang_lin19@mails.jlu.edu.cn (L.Y.); yuanzhu16@mails.jlu.edu.cn (Y.Z.); xiaowy21@mails.jlu.edu.cn (W.X.); wangzr20@mails.jlu.edu.cn (Z.W.); xiaochuntang@jlu.edu.cn (X.T.)

**Keywords:** ASFV, PRRSV, PEDV, CSFV, PRV, TGEV, antiviral pigs, host factors, CRISPR/Cas9 library

## Abstract

Pigs play an important role in agriculture and biomedicine. The globally developing swine industry must address the challenges presented by swine-origin viruses, including ASFV (African swine fever virus), PRRSV (porcine reproductive and respiratory syndrome virus), PEDV (porcine epidemic diarrhea virus), PRV (pseudorabies virus), CSFV (classical swine fever virus), TGEV (transmissible gastroenteritis virus), et al. Despite sustained efforts by many government authorities, these viruses are still widespread. Currently, gene-editing technology has been successfully used to generate antiviral pigs, which offers the possibility for increasing animal disease tolerance and improving animal economic traits in the future. Here, we summarized the current advance in knowledge regarding the host factors in virus infection and the current status of genetically modified pigs that are resistant to virus infection in the world. There has not been any report on PEDV-resistant pigs, ASFV-resistant pigs, and PRV-resistant pigs owing to the poor understanding of the key host factors in virus infection. Furthermore, we summarized the remaining problems in producing virus-resistant pigs, and proposed several potential methods to solve them. Using genome-wide CRISPR/Cas9 library screening to explore the key host receptors in virus infection may be a feasible method. At the same time, exploring the key amino acids of host factors in virus infection with library screening based on ABEs and CBEs (Bes) may provide creative insight into producing antiviral pigs in the future.

## 1. Introduction

Pigs, as one of the most important types of livestock, play an indispensable role in agriculture. They share similar genetic, physiological, and anatomical features and body sizes with humans, and are regarded as important candidates for organ donors for xenotransplantation. Furthermore, pigs are an important model organism for insights into the mechanisms of human disease [1,2,3]. Hence, it is critical to maintain the stability of the swine industry for the benefits of the agricultural and biomedicine industries. To this end, the globally developing swine industry must address the challenges represented by swine-origin viruses, including ASFV, PRRSV, PEDV, PRV, CSFV, TGEV, et al.

**Figure 1:**
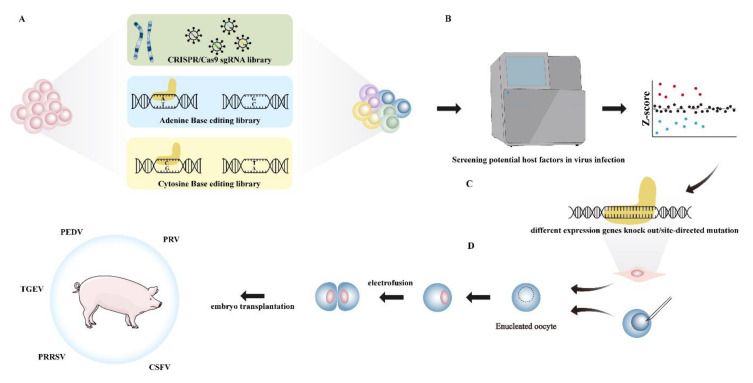
Possible method of producing virus-resistant pigs in the future. (**A**) First, genome-wide knockout cells were constructed from lentivirus-packaged CRISPR/Cas9 library or ABE/CBE library. (**B**) Then, candidate host factors were enriched and screened by next-generation sequencing technology after several rounds of viral infection. (**C**) Fetal fibroblast cells over-expressing or without candidate host factors were prepared. (**D**) Then, the donor cells were injected into the enucleated oocytes, and cloned pigs were prepared by embryo transplantation.

In recent decades, vaccines against porcine viral diseases have been developed to enhance the adaptive immunity of hosts [4,5]. However, several viruses can still escape immune surveillance [6,7,8,9,10]. Accordingly, it is of great importance to develop an efficient means to protect hosts from being infected by these viruses or to block virus replication. Recently, gene-editing technology, such as CRISPR/Cas9, ABEs (Adenine Base Editors), CBEs (Cytosine Base Editors), and prime editing, has been successfully applied in pigs to increase animal disease tolerance and improve economic traits. Therefore, generating genetically modified pigs with gene-editing technology may be another feasible method to fight against swine-origin viruses. The focuses of this review are the current status of virus-resistant pigs in the world and the existing problems. In addition, we put forward two possible solutions to the problems: identifying the key host receptors in virus infection with genome-wide CRISPR/Cas9 library screening, and exploring the key amino acids of host factors in virus infection with BE-induced library screening (Figure 1).

## 2. Current Progress of Genetically Modified Pigs That Are Resistant to CSFV Infection

Classical swine fever (CSF), one of the most highly contagious swine diseases, characterized by high fever and high mortality, is caused by the classical swine fever virus, and leads to tremendous economic losses to the swine industry throughout the world [11,12]. The classical swine fever virus belongs to the *Pestivirus* genus within the *Flaviviridae* family, and is an enveloped and positive-sense RNA virus [13]. According to the sequence of virus genomes, CSFV was classified into three genotypes (genotypes 1 to 3) and 11 sub-genotypes (1.1 to 1.4, 2.1 to 2.3, and 3.1 to 3.4) [14,15]. It was reported that the CSFV strain of genotype 2 was the dominant strain in the world, while the other two strains are also epidemic [16,17]. 

There are 38 CSFV-free regions in the world according to the World Organization for Animal Health (OIE), which are mainly located in North America, the European Union, Oceania, Asia, Eastern Europe, and part of Africa. Furthermore, it was reported that CSFV recurred in a supposedly CSFV-free country, Japan, due to a reservoir of CSFV in wild boars in that country [18]. Therefore, as an endemic and recurring porcine virus, CSFV is still a considerable factor affecting the porcine industry, especially in China. 

Currently, live attenuated vaccines are the most widely used strategy to control CSFV in the world. Classical CSF vaccines, such as c-strain, GPE-strain, LPC-strain, and LK-VNIVViM strain, provided robust protection from CSFV infection [19], though they lack DIVA (differentiating infected from vaccinated animals) capacity. Thus, several marker DIVA vaccines were developed, such as FlagT4Gv, TWEJ2, Flc-LOM-BE^rns^, and other E2 subunit DIVA vaccines [20]. 

Despite consistent efforts by many government authorities, it is still difficult to stamp out CSFV in infected areas and re-emerging areas. The live attenuated vaccines and the marker DIVA vaccines result in subclinical infection and immunosuppression, which makes it even harder to eliminate CSFV. As a result, another strategy is urgently needed to control the virus in pigs.

### 2.1. Host Factors in CSFV Infection

A number of host factors were identified to have participated in the process of CSFV replication and pathogenesis (Table 1). Several of these host factors were found to have antiviral activity, the over-expression of which could inhibit CSFV growth, and the knock down of which promoted CSFV growth. The others worked on CSFV growth, where knocking them down promoted virus growth. Since these hosts are the potential targets that offer new prospects for developing antiviral strategies, more and more researchers are concentrating on generating anti-CSFV pigs with these targets. 

### 2.2. Progress of Genetically Modified Pigs That Are Resistant to CSFV Infection

Several host factors with anti-CSFV activity were utilized to generate anti-CSFV pigs. In 2016, our group generated pigs that over-express MxA [44]. Later, the *Rosa26* site-specific integration pigs of RSAD2 were produced in our lab [45]. All these pigs exhibited the ability to inhibit CSFV growth.

In addition, RNAi has been regarded by virologists as a promising way to suppress virus infection. To date, there have been several RNAi-based studies on CSFV suppression in vitro, and these studies have indicated that the development of shRNA-TG pigs that are resistant to CSFV may be possible [46]. Anti-CSFV shRNA was integrated specifically into porcine *Rosa26* sites and porcine *miR-17-92* clusters in our lab [47,48]. The viral challenge assays demonstrated that these TG pigs could effectively limit the replication of CSFV and reduce clinical signs and mortality. Moreover, they could be stably transmitted to F1 generation (Table 2). 

## 3. Current Progress of Genetically Modified Pigs That Are Resistant to ASFV Infection

African swine fever (ASF), which is caused by the African swine fever virus (ASFV), is a hemorrhagic and infectious disease listed by the OIE, causing enormous economic losses each year [49]. ASFV belongs to the *Asfarviridae* family, and contains a linear double-strand DNA. The genome of ASFV is about 170 kb-190 kb and encodes about 151–167 ORFs [50]. Currently, there has been no efficient vaccine against ASF. The main and efficient strategies to control ASF are quarantine and slaughtering the infected pigs. 

Like porcine reproductive and respiratory syndrome virus (PRRSV), ASFV replicates predominantly in porcine alveolar macrophages (PAMs). Previous studies showed that ASFV mainly replicates in specific cytoplasmic sites, which have been referred to as viral factories, albeit a lot of ASFV DNA synthesis takes place in the nucleus in the early stages of infection. Despite decades of efforts by virologists, the key membrane receptor in the process of ASFV entry has not been identified yet. Early studies implied that ASFV entry into host cells is through receptor-mediated endocytosis [51,52]. Given the cell tropism of ASFV, several macrophage membrane receptors, including CD163, MHC II, CD203a, and CD45, were under suspicion as important molecules in ASFV infection [53]. In 2003, researchers demonstrated that ASFV infects CD163^+^ monocyte subpopulations, but not CD163^-^ monocyte subpopulations. Blocking the membrane receptor of primary alveolar macrophages with mAbs 2A10 and 4E9 inhibits ASFV infection, suggesting that CD163 acts as an important membrane receptor in the process of ASFV infection [54]. However, it was confirmed that CD163 was not the receptor of ASFV, and there was no difference in clinical signs and survival rates between CD163-knockout pigs and control pigs after ASFV challenge [55]. So far, few key host factors have been identified owing to cell tropism and the limited technology for host factor screening, albeit several host factors, such as EGFR (epidermal growth factor receptor), PI3Ks (phosphoinositide 3-kinases), PAK1 (p21-activated kinase-1), NPC1 (Niemann-Pick C1), and NPC2 (Niemann-Pick C2), were found to be involved in ASFV entry [56,57,58]. 

It was announced by Chinese scientists in 2020 that Lansibai-2 pigs (LS-2), which were bred by China Shandong Landsee Genetics Co., Ltd., were resistant to ASFV. Previous results implied that LS-2 pigs showed significant ASFV resistance following oral challenge with an SY18 strain at the dosage of 10^6.0^ TCID50. Five out of six LS-2 pigs were found to have low viremia at 9 dpi, while the common domestic pigs were found to have fever and viremia at 3 dpi. In the end, five out of six LS-2 pigs survived, and all the common domestic pigs died at less than 10 dpi [59]. 

More attention should be paid to exploring key receptors with CRISPR/Cas9 library screening technology in the future. Additionally, generating anti-ASFV pigs with functionally annotated gene targets may be a promising strategy to control ASFV.

## 4. Current Progress of Genetically Modified Pigs That Are Resistant to PRRSV Infection

Porcine reproductive and respiratory syndrome, which is caused by the porcine reproductive and respiratory syndrome virus (PRRSV), is an economically significant contagious disease [60]. PRRSV is a small, enveloped, positive-sense single-strand RNA virus, belonging to the *Arteriviridae* family in the order *Nidovirales* [61]. The genomes of PRRSV are approximately 15 kb in size, which encode at least ten ORFs. There are two well-characterized genotypes: type 1, also known as European-like (EU-type); type 2, also known as Northern American-like (NA-type) [62]. In China, the most common PRRSV isolate was NA-type, which was first reported in 1996. In 2006, a highly pathogenic PRRSV named HP-PRRSV was first identified in China, and an HP-PRRSV epidemic in China caused enormous losses in the Chinese swine industry [63]. Recombination and mutation are the main strategies of PRRSV evolution, and they play an important role in increasing PRRSV variation. In 2013, a new, recombined PRRSV, termed NADC30, which was like PRRSV, was isolated in China [64].

Vaccination has been the major strategy to prevent PRRSV infection in the past two decades. Multiple modified live and inactivated vaccines against both types of PRRSV have been developed to control PRRSV transmission. However, these vaccines failed to provide sustainable protection owing to the high recombination and variation of PPRSV, which delayed the neutralization antibody response and led to antibody-dependent enhancement (ADE) to counter host immunity [65,66]. Accordingly, PRRS has been one of the most important animal diseases. Therefore, other effective multiple heterologous PRRSV protection methods are urgently needed. The CRISPR/cas9-based gene editing of the key host factors which interact with PRRSV is a promising prospect. Here, we review the latest progress in identifying host factors that interact with PRRSV and anti-PRRSV pigs across the world. 

### 4.1. CD163 and Other Host Factors in PRRSV Infection

Numerous studies have shown that PRRSV entry is mediated by various cell receptors, such as sialoadhesin (Sn, CD169) [67,68], CD151 [69,70], heparin sulfate [71], vimentin [72], MYH9 [73,74], and CD163 (a scavenger receptor) [75]. Recently, CD163 was reported to be the key receptor during the process of PRRSV infection [76]. The knock out of CD163 inhibited PRRSV infection, and the over-expression of CD163 in the membranes of PRRV non-permissive cells converted them to PRRSV permissive cells [77]. CD163, known as a scavenger receptor, is a type 1 transmembrane glycoprotein, which is expressed on the surface of monocyte/macrophage lineages (such as PAMs) and MARC145 cells [78]. It was announced that CD163 consists of nine scavenger receptor cysteine-rich (SRCR) domains in the extracellular domain (SRCR1-SRCR9), among which the SRCR2 domain was shown to support the adhesion of erythroblastic cells, facilitating their maturation into erythrocytes, while SRCR3 was shown to clear the free hemoglobin within the plasma, and SRCR5 was shown to be essential to PRRSV entry [79]. Knocking out porcine CD163 SRCR5, or replacing it with human CD163L1 SRCR10, made it resistant to PRRSV [75,79].

Besides these PRRSV entry blockers, some host factors that act in PRRSV infection were found, too. (Table 3). Jiang et al. [80] found that ZAP, a zinc finger antiviral protein, interacts with the N-terminal amino acids (150–160 aa) of NSP 9, and acts as an antiviral host factor to prevent PRRSV replication. Guo et al. [81] reported that triggering receptor expression on myeloid cells 2 (TREM2), which includes dendritic cells and macrophages, interacts with NSP 2 to promote PRRSV replication. Silencing TREM2 significantly inhibits PRRSV replication, and the over-expression of TREM2 promotes PRRSV replication in PAMs. In addition, USP18 [82], LSM14A [83], heme oxygenase-1 [84], cholesterol 25-hydroxylase [85], and MOV10 [86] were verified to play a negative role in PRRSV replication; nevertheless, DDX18 [87], Rab11a [88], and poly (C)-binding protein 1 and 2 [89] play a positive role in PRRSV replication.

### 4.2. Current Progress of Genetically Modified Pigs That Are Resistant to PRRSV Infection

In 2013, Li et al. [93] reported that PRRSV replication was inhibited in transgenic pigs expressing PRRSV-specific shRNA in vitro and in vivo. The result of PRRSV challenge demonstrated the transgenic pigs exhibited reduced serum PRRSV titers compared with wild-type pigs. Additionally, the transgenic pigs survived 11 days, while the wild-type pig survived 8 days. Sialoadhesin (Sn), also known as Siglec1 or CD169, is a macrophage-restricted molecule in the immunoglobulin (Ig) superfamily and a type I transmembrane (TM) glycoprotein [94]. CD169 has been extensively studied as an essential receptor for PRRSV infection by mediating the capture and internalization of the virus [95]. Furthermore, Prather et al. [96] announced that CD169-knockout pigs exhibited no significant PRRSV resistance, and CD169 was not required in the PRRSV infection. Most importantly, Whitworth [97] first verified in 2015 that CD163-knockout pigs showed significant PRRSV resistance, and the CD163-gene-modified pigs experienced no viremia and no clinical signs. Hereafter, many CD163-knockout pigs were generated with CRISPR/Cas9, and all the CD163-modified pigs showed resistance to type 1 and type 2 PRRSV [98,99,100,101]. Besides, it was acknowledged that substituting porcine CD163 SRCR5 with human CD163L1 SRCR10 conferred resistance to PRRSV to pigs [102]. Wells et al. and Li et al. produced gene-edited pigs by substituting porcine CD163 SRCR5 with human CD163L1 SRCR10 [103,104]. The results of virus challenge in vivo from Wells et al. showed that these pigs were resistant to type 1 PRRSV but not to type 2 PRRSV. However, Li et al. demonstrated that these pigs showed highly pathogenic porcine reproductive and respiratory syndrome virus (HP-PRRSV) resistance. 

Accordingly, CD163-gene-edited pigs with great PRRSV resistance exhibit fine prospects for controlling viral infection and also lay the foundation for controlling others virus. Nevertheless, there are still some concerns that need to be addressed. Firstly, CD163 plays an important role in vivo. It remains unknown whether knocking out CD163 SRCR5 affects the function of CD163. Additionally, it is still an issue of concern as to whether substituting CD163 SRCR5 with human CD163 L1 SRCR10 renders humans sensitive to PRRSV. Hence, exploring the key amino acids that function in the process of PRRSV infection in the CD163 may be another approach to address these concerns. 

## 5. Current Progress of Virus-Resistant Pigs in Porcine Enteric Coronaviruses and the Other Viruses

Porcine enteric coronaviruses (PECs) cause high mortality and morbidity in newborn piglets. Such viruses include porcine epidemic diarrhea virus (PEDV), transmissible gastroenteritis virus (TGEV), swine acute diarrhea syndrome coronavirus (SADS-CoV), and porcine delta coronavirus (PDCoV) [105]. It was announced that PECs could potentially transmit into humans and cause enormous economic losses in the pig industry in China and around the world. In China, several attenuated and inactivated vaccines were applied to control PECs [106,107]. However, their effectiveness is still debated due to the unique characters of PECs, such as the low oral infectious dose and the ineffective immunogenicity of vaccines [108]. Therefore, an effective and safe method is urgently needed to control these PECs.

Porcine aminopeptidase-N (pAPN), which is mainly expressed on the surface of enterocytes, was first reported to serve as a receptor of TGEV in 1992 [109]. Later, it was verified that pAPN was also an important receptor of PDCoV [110,111,112]. The overexpression of pAPN in non-permissive cell lines rendered it susceptible to TGEV and PDCoV. Knocking out pAPN in swine testis cells (ST cells) significantly decreased TGEV and PDCoV attachment, but not PEDV [113]. Hence, pAPN, which acts as an important receptor of TGEV and PDCoV, may be a potential gene target to produce TGEV-resistant pigs. In 2019, Whitworth et al. [114] generated pAPN-null pigs with CRISPR/Cas9. The results of virus challenges showed that pAPN-null pigs were resistant to TGEV, but not to PEDV. Furthermore, Li et al. [100] produced CD163 and pAPN-double-knockout pigs in 2020. It was the first report on gene-edited pigs with PRRSV, TGEV, and PDCoV resistance simultaneously, while maintaining the same growth and reproductive production traits compared with wild-type pigs.

Similarly, Ouyang et al. [115] produced transgenic pigs expressing shRNA directed to foot-and-mouth disease virus (FMDV) VP1 sequences. Upon FMDV challenge, transgenic pigs remained non-febrile and showed lower viremias and clinical scores compared to wild-type pigs. Furthermore, RSAD2 is a member of the radical S-adenosylmethionine (SAM) superfamily of enzymes [116]. Many reports have shown that RSAD2 exhibits antiviral activity against a broad range of viruses, including influenza A, Zika virus, and so on [117]. A recent study indicated that pRSAD2 effectively inhibits CSFV replication in vitro via the interaction with the CSFV E2 protein [118]. Xie et al. [45] found that the pRSAD2 knock in (pRSAD2 KI) of the *pRosa26* locus of PK-15 cells made it resistant to CSFV and PRV, but the resistance of pRSAD2 KI pigs to CSFV and PRV infections was undetermined. At the same time, it was reported that human CCCH-type zinc finger proteins containing the 11A protein (ZC3H11A) are essential for the replication of multiple nuclear-replicating viruses (such as HIV, influenza virus, herpes simplex virus, and adenovirus) in human cells [119], which suggested porcine ZC3H11A may be an ideal gene target to prevent PRV, PCV, and even ASFV in the future.

## 6. Concluding Remarks

At present, there are many pigs resistant to CSFV, PRRSV, and TGEV being produced, which suggested the enormous potential of CRISPR/Cas9-based gene-editing technology for shortening the breeding cycle and enhancing disease tolerance in pigs. However, there have been no reports of pigs resistant to PEDV, ASFV, and PRV owing to the lack of understanding of the key receptors in viral infection. In the future, genome-wide CRISPR/Cas9 library screening technology may be an ideal method to explore the key receptors in PEDV, ASFV, and PRV infection. 

Gene-edited pigs showed remarkable virus resistance, indicating a fine prospect for controlling virus infection. Nevertheless, there are still some concerns emerging. First, it was reported that CRISPR/Cas9 may induce megabase-scale chromosomal truncations in cell lines and primary cells with P53-dependent mechanisms [120]. Some host factors, such as CD163, play an important role in vivo, but whether knocking out CD163 SRCR5 affects the function of CD163 is not yet understood. In addition, the types and population of gene-edited pigs are limited. It is essential to increase the population size and supervise the production traits and reproduction traits of gene-edited pigs. Additionally, it is still unknown whether substituting porcine receptor proteins with homologous proteins from other species can make the virus contagious to other species. Hence, exploring the key amino acids within the key host receptor that acts in the process of virus infection may be another desirable solution to these concerns in the future.

## Figures and Tables

**Table 1:** Host factors that act in CSFV infection.

Host Factors	Function in CSFV Infection	Promote (+) or Inhibit (−) CSFV Growth in Host Cell	Reference
Annexin2	Interacts with CSFV E2 and NS5A, promote CSFV replication	+	[21,22]
IFITM1-3 (interferon-induced trans membrane protein 3)	Modifies the membrane structure or alter endosomal physiology to impair viral membrane fusion	−	[23]
ARFGAP1 (ADP-ribosylation factor GTPase-activating protein 1)	Binds to CSFV NS5A and promote CSFV replication	+	[24]
β-actin	The amino acids 95-188 of β-actin are responsible for the interaction between β-actin and CSFV E2	+	[25]
Caveolin-1	CAV1-mediated endocytosis is necessary for CSFV invasion	+	[26]
NDP52 (nuclear dot protein 52)	CSFV inhibits NDP52 expression. Additionally, inhibiting NDP52 promotes interferon and TNF release, acting on the NF-κB pathway	+	[27]
GBP1 (guanylate-binding protein 1)	The N-terminal globular GTPase domain of GBP1 interacts with CSFV NS5A. Overexpression of GBP1 inhibits CSFV replication; knocking down GBP1 significantly promotes CSFV replication. Furthermore, the K51 of GBP1 is essential for CSFV replication	−	[28]
PSMB10 (proteasome subunit beta 10)	Acts as an NS3-interacting partner in CSFV infection. Overexpression of PSMB10 inhibited CSFV replication	+	[29]
POASL (interferon-inducible oligoadenylate synthetase-like protein)	Interacts with MDA5 to enhance MDA5-mediated type I IFN signaling and suppress CSFV replication	−	[30]
MERTK (Mer tyrosine kinase)	Interacts with CSFV E2 to facilitate CSFV entry, and down-regulates the expression of IFN-β to enhance CSFV replication	+	[31]
MG132	Activates JAK-STAT pathway and up-regulates several interferon-stimulated genes’ (ISGs) expression in CSFV infection cells	−	[32]
RACK1 (receptor for activated C kinase 1)	RACK1 interacts with NS5A, inhibiting CSFV replication by inhibiting NF-κB activation	−	[33]
PRNF114 (porcine RING finger protein 114)	Interacts with NS4B and degrades NS4B through a proteasome-dependent pathway	−	[34]
Rab1b, Rab5, Rab7, and Rab11	Regulates CSFV endocytosis	+	[35,36]
Rab18	Interacts with NS5A and mediates virus replication and assembly	+	[37]
DCNT6 (dynactin subunit 6)	Interacts with E2, and the DCNT6-E2 interaction is important for CSFV replication and viral virulence	+	[38]
Torsin-1A	Interacts with E2, disrupting Torsin-1A-E2 interaction to completely inhibit CSFV replication	+	[39]
CCDC115 (coiled-coil domain-containing 115)	CCDC115-E2 interaction is essential for CSFV replication in swine macrophages	+	[40]
LamR (laminin receptor)	Acts as an alternative attachment receptor, interacting with E^rns^	+	[41]
Fatty acid synthase (FASN)	FASN participates in the formation of the replication complex. Knocking down FASN in host cells inhibits CSFV replication	+	[42]
PCBP1 (poly C-binding protein 1)	Interacts with N^pro^, down-regulating type I interferon in CSFV infection cells	+	[43]

**Table 2:** Advance of genetically modified pigs resistant to CSFV infection.

Genotype	Country	Institution	Research Group	Reference
Anti-CSFV shRNA	China	Jilin University	Ouyang group	[47,48]
RADS2 knock-in	China	Jilin University	Ouyang group	[45]
MxA overexpression	China	Jilin University	Ouyang group	[44]

**Table 3:** Host factors that act in PRRSV infection.

Host Factors	Function in PRRSV Infection	Promote (+) or Inhibit (−) PRRSV Growth in Host Cell	Reference
Sn	The first 150 amino acids of the Sn N-terminal region are essential for the attachment of PRRSV	+	[67]
CD151	Interacts with PRRSV 3 UTR RNA; knocking down CD151 in Marc-145 cells significantly suppresses PRRSV infection	+	[70]
MYH9 (myosin heavy chain 9)	Interacts with GP5 via its C-terminal domain and confers cells susceptible to PRRSV	+	[90]
Vimentin	Acts as a virus receptor, leading to the opsonization and endocytosis of PRRSV	+	[72,91]
CD163	Acts as a key receptor; CD163 interacts with GP2a and GP4. CD163 SRCR5 plays an important role in PRRSV infection, and deleting SRCR5 inhibits PRRSV proliferation	+	[75]
ZAP (zinc finger antiviral protein)	Interacts with NSP9, and acts as an efficient antiviral host factor to inhibit PRRSV infection	−	[80]
TREM2 (triggering receptor expressed on myeloid cells 2)	Down-regulating TREM activates the PI3K/NF-κB signal pathway, reinforcing the expression of proinflammatory cytokines and type I interferons	+	[81]
USP18	Alternates the nuclear translocation of NF-ΚB P65 and p50; the overexpression of USP18 restricts PRRSV growth	−	[82]
DDX18	Interacts with NSP2 and NSP 10; silencing DDX18 inhibits PRRSV replication	+	[87]
LSM14A	Up-regulates the activities of IFN-β and ISRE promoters, enhancing IFN-β, RIG-1, and ISGs expression; inhibits the expression of TNF-α and IL-6	−	[83]
Heme oxygenase-1	Generates down metabolite CO, and suppresses PRRSV replication by activating the cyclic cGMP/PKG signal pathway	−	[84]
Rab11a	Acts as a pro-viral host factor in PRRSV replication and plays a vital role in autophagosome maturation	+	[88]
Poly (C)-binding protein 1 and 2	Binds to the 5 UTR of PRRSV, silencing PCBP1 and PCBP2 and inhibiting PRRSV replication	+	[89]
Cholesterol 25-hydroxylase	Restricts PRRSV replication by targeting viral penetration, as well as degrading NSP1α and silencing CH25H, to promote PRRSV replication	−	[85]
MoV10 (Moloney leukemia virus 10-like protein)	Interacts with N proteins and affects the distribution of N proteins in the cytoplasm and nucleus, leading to the retention of N proteins	−	[86]
Sydecan-4	Mediates PRRSV entry by interacting with EGFR	+	[92]

## Data Availability

All data generated or analyzed during this study are included in this published article.

## Abbreviations

**ABE**	Adenine base editor
**ADE**	Antibody-dependent enhancement
**ASFV**	African swine fever virus
**CBE**	Cytosine base editor
**CSFV**	Classical swine fever virus
**FMDV**	Foot-and-mouth disease virus
**HCV**	Hepatitis C virus
**HP-PRRSV**	Highly pathogenic porcine reproductive and respiratory syndrome
**HIV**	Human immunodeficiency virus
**pAPN**	Porcine aminopeptidase-N
**PDCoV**	Porcine delta coronavirus
**PECs**	Porcine enteric coronaviruses
**PEDV**	Porcine epidemic diarrhea virus
**PRRSV**	Porcine reproductive and respiratory syndrome
**PRV**	Pseudorabies virus
**ST Cells**	Swine testis cells
